# Targeting the cGAS-STING Pathway to Modulate Immune Inflammation in Diabetes and Cardiovascular Complications: Mechanisms and Therapeutic Insights

**DOI:** 10.3390/cimb47090750

**Published:** 2025-09-12

**Authors:** Guida Cai, Xi Zhang, Jiexi Jiao, Weijie Du, Meiling Yan

**Affiliations:** 1 Guangdong Metabolic Diseases Research Center of Integrated Chinese and Western Medicine, Key Laboratory of Glucolipid Metabolic Disorder, Ministry of Education of China, Guangdong Key Laboratory of Metabolic Disease Prevention and Treatment of Traditional Chinese Medicine, Institute of Chinese Medicine, Guangdong Pharmaceutical University, Guangzhou 510006, China; 2 State Key Laboratory of Frigid Zone Cardiovascular Diseases (SKLFZCD), Department of Pharmacology (State-Province Key Laboratories of Biomedicine-Pharmaceutics of China, Key Laboratory of Cardiovascular Research, Ministry of Education), College of Pharmacy, Harbin Medical University, Harbin 150081, China

**Keywords:** type 2 diabetes mellitus (T2DM), diabetic cardiovascular complications, immunological mediators, inflammatory responses, cGAS-STING signaling pathway

## Abstract

Type 2 diabetes mellitus (T2DM), characterized by insulin resistance and chronic hyperglycemia, markedly increases the incidence and mortality of cardiovascular disease (CVD). Emerging preclinical evidence identifies the cyclic GMP-AMP synthase-stimulator of interferon genes (cGAS–STING) pathway as a critical mediator of diabetic cardiovascular inflammation. Metabolic stressors in T2DM—hyperglycemia, lipotoxicity, and mitochondrial dysfunction—induce leakage of mitochondrial and microbial double-stranded DNA into the cytosol, where it engages cGAS and activates STING. Subsequent TBK1/IRF3 and NF-κB signaling drives low-grade inflammation across cardiomyocytes, endothelial cells, macrophages, and fibroblasts. Genetic deletion of cGAS or STING in high-fat-diet-fed diabetic mice reduces NLRP3 inflammasome-mediated pyroptosis, limits atherosclerotic lesion formation, and preserves cardiac contractile performance. Pharmacological inhibitors, including RU.521 (cGAS antagonist), C-176/H-151 (STING palmitoylation blockers), and the TBK1 inhibitor amlexanox, effectively lower pro-inflammatory cytokines (IL-1β, IL-6, TNF-α) and improve left ventricular ejection fraction in diabetic cardiomyopathy and ischemia–reperfusion injury models. Novel PROTAC degraders targeting cGAS/STING and natural products such as Astragaloside IV and Tanshinone IIA further support the pathway’s druggability. Collectively, these findings position the cGAS–STING axis as a central molecular nexus linking metabolic derangement to cardiovascular pathology in T2DM and underscore its inhibition or targeted degradation as a promising dual cardiometabolic therapeutic strategy.

## 1. Introduction

Diabetes mellitus (DM) stands as a major challenge in global public health in the 21st century, with its disease burden growing exponentially. According to the International Diabetes Federation’s 2021 report, the number of adults aged 20–79 with diabetes worldwide has reached 537 million, with type 2 diabetes mellitus (T2DM) accounting for over 90% [1]. Notably, epidemiological models predict that the number of patients will soar to 783 million by 2045, highlighting the urgency of improving the diabetes prevention and control system. Among the multi-system complications caused by diabetes, cardiovascular diseases (CVD), due to their high fatality rate, have become a key and difficult point in clinical management [2].

Recent studies reveal that cytosolic double-stranded DNA (dsDNA) sensing and the ensuing innate immune response play pivotal roles in diabetic CVD. The cyclic GMP-AMP synthase (cGAS)–stimulator of interferon genes (STING) axis serves as a primary cytosolic DNA sensor, activating downstream TANK-binding kinase 1 (TBK1)/(Interferon Regulatory Factor 3) IRF3 and Nuclear factor-kappa B (NF-κB) pathways to induce type I interferons (IFN-I) and pro-inflammatory cytokines, thereby driving vascular inflammation, myocardial fibrosis, and remodeling [3,4]. Although several reviews have examined cGAS-STING signaling in metabolic disorders, sterile inflammation, and cardiac injury [5,6,7], they predominantly focus on antiviral or tumor-related contexts and lack a systematic integration of T2DM-specific pathophysiological features—such as hyperglycemia-induced mitochondrial dysfunction, lipotoxicity, and gut dysbiosis—and their cell-type–specific activation within the cardiovascular system. Yet, cGAS–STING is of particular relevance in T2DM, since metabolic stressors (e.g., hyperglycemia, lipid overload, mitochondrial damage) trigger mitochondrial and nuclear DNA leakage, resulting in chronic cGAS-STING activation and low-grade inflammation that exacerbates vascular and myocardial injury. This review is organized as follows: (1) Mechanistic Overview: DNA leakage-driven cGAS–STING activation in diabetic tissues, including mtDNA and microbial DNA translocation. (2) Spatial Regulation: Influence of mitochondrial stress, organelle crosstalk, and epigenetic modulators on the spatiotemporal dynamics of cGAS–STING signaling. (3) Therapeutic Strategies: Current progress in small-molecule inhibitors, targeted degraders, and natural compounds aimed at modulating the cGAS–STING axis. Through this structured analysis, we aim to clarify established mechanisms, identify critical knowledge gaps, and propose directions for precision targeting of the cGAS–STING pathway in diabetic cardiovascular disease.

## 2. cGAS-STING Pathway

The cGAS-STING pathway can be broadly divided into two branches: the canonical and non-canonical pathways. The canonical pathway primarily involves cGAS, STING, and downstream effectors such as TBK1 and IRF3, leading to the production of type I interferons. In contrast, the non-canonical pathway includes alternative STING-mediated signaling mechanisms that may function independently of TBK1 or IRF3. In the following section, we provide a detailed overview of both branches.

### 2.1. Canonical cGAS-STING Pathway

**Figure 1:**
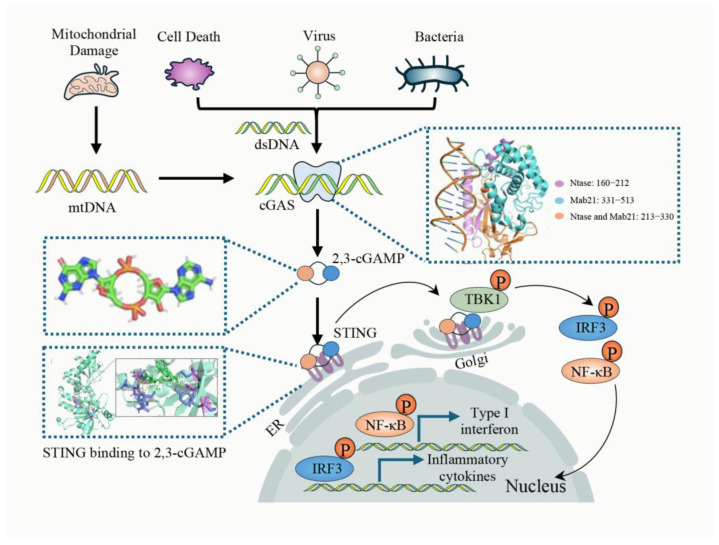
Canonical cGAS-STING pathway. cGAS is a key immune-sensing enzyme that detects cytosolic DNA and triggers immune responses. Its active domains, including DNA-binding and catalytic regions, are mainly in the C-terminal part. Upon dsDNA recognition, cGAS changes conformation and catalyzes ATP and GTP into 2′,3′-cGAMP. This second messenger binds to and activates STING, inducing its translocation from the endoplasmic reticulum to the Golgi apparatus. There, STING activates TBK1, which phosphorylates STING, activating transcription factors like IRF3 and NF-κB. Protein structure data were acquired from the RCSB PDB [20], and the ligand’s chemical structure was obtained via the PubChem database [21]. Molecular docking between human STING and 2′,3′-cGAMP was performed using AutoDock 4.2.6. The resulting complex was visualized and refined using PyMOL 2.5.0 to improve structural representation.

cGAS is a key nucleic acid sensor enzyme that detects cytoplasmic DNA in mammals. Upon binding DNA, cGAS synthesizes cyclic GMP-AMP (cGAMP), which activates STING and subsequently triggers the secretion of type I interferons and other cytokines to mediate immune responses [8]. Structurally, cGAS features an N-terminal DNA-binding site, a central catalytic domain, and a C-terminal Mab-21 domain, which belongs to the MAB21 protein family. The core catalytic domain (residues 160–330) facilitates substrate binding and catalyzes cGAMP synthesis by coordinating two magnesium ions (Mg^2+^), while the Mab21 domain (residues 213–513) enhances DNA binding and promotes cGAS dimerization, further boosting catalytic activity [9,10,11]. cGAS recognizes dsDNA through three key binding sites identified by X-ray crystallography: Site A, which interacts with the DNA backbone to initiate activation; Site B, which stabilizes the cGAS-DNA complex through hydrogen bonds and electrostatic interactions; and Site C, located at the C-terminus, which promotes cGAS dimerization and enhances DNA binding, thereby increasing catalytic activity [6]. This coordinated interaction enables cGAS to detect cytoplasmic DNA, triggering immune responses crucial for antiviral and antibacterial defense [12]. In its inactive state, cGAS is poised to detect free dsDNA, which may derive from various sources, including viruses, bacteria, dead cells, or mitochondrial damage that releases mitochondrial DNA (mtDNA). Upon interaction with dsDNA, cGAS undergoes a structural rearrangement, leading to the activation of its catalytic activity. This activated cGAS catalyzes the conversion of GTP and ATP into the cyclic dinucleotide cyclic GMP-AMP (cGAMP), a second messenger within the cell [13,14]. As an essential intracellular second messenger, cGAMP engages directly with STING (stimulator of interferon genes), a key transmembrane adaptor protein anchored in the endoplasmic reticulum (ER), which functions as the central signaling platform of the innate immune response. Upon ligand binding, STING undergoes a conformational alteration that facilitates its oligomer formation and mobilization from the ER via vesicles coated with coat protein complex II. Once trafficked to the Golgi apparatus, STING is modified through palmitoylation at evolutionarily conserved cysteine sites—namely Cys88 and Cys91—a post-translational event indispensable for its complete activation and for recruiting downstream signaling molecules. [15,16]. At the Golgi, STING serves as a molecular scaffold, enabling the docking and activation of TANK-binding kinase 1 (TBK1). TBK1 becomes fully enzymatically active upon trans-autophosphorylation at serine residue 172. Activated TBK1 subsequently phosphorylates IRF3 at serine residues within its carboxy-terminal domain. The phosphorylated IRF3 dimerizes and translocates into the nucleus, where it binds to interferon-stimulated response elements located in the promoter regions of type I interferon genes, including IFN-β, thereby initiating transcription of antiviral effectors. Concurrently, STING signaling activates the IκB kinase (IKK) complex, particularly IKKβ, which phosphorylates the inhibitor of NF-κB. This phosphorylation marks IκBα for ubiquitination and subsequent proteasomal degradation, thereby liberating the NF-κB p65/p50 heterodimer. Freed NF-κB translocates into the nucleus, where it binds κB motifs in the promoters of multiple pro-inflammatory cytokines, including tumor necrosis factor-alpha (TNF-α), interleukin-6 (IL-6), and interleukin-1β (IL-1β), amplifying the inflammatory response [14,17]. Moreover, the activation of IRF3 or NF-κB triggers a signaling cascade that facilitates the formation of the NLRP3 inflammasome complex, which consists of NLRP3, ASC, and caspase-1, governed by NLRP3 transcription. Caspase-1 subsequently cleaves gasdermin D (GSDMD), producing an active N-terminal fragment that creates pores in the cell membrane, ultimately triggering pyroptosis. Pyroptosis results in the release of inflammatory mediators, such as IL-1β and (L-18), into the extracellular environment, hence intensifying the inflammatory response [18,19]. The classical cGAS–STING signaling pathway is illustrated in Figure 1.

### 2.2. Non-Canonical cGAS-STING Pathway

STING activation is not limited to the canonical pathway but also involves non-canonical pathways, such as the STING-PERK-eIF2α signaling axis, which plays a critical role in cellular senescence and organ fibrosis. Upon association with cGAMP, STING engages with protein kinase R-like endoplasmic reticulum kinase (PERK), directly activating it. PERK then phosphorylates eIF2α at Ser51, resulting in a global suppression of mRNA translation within the cell [22].

## 3. The cGAS-STING Pathway and T2DM

As a critical DNA sensor for interferon inflammatory response, cGAS and its downstream effect signal pathways are central to the cardiovascular complications induced by metabolic stress in T2DM [23]. Beyond its role in pathogen recognition, cGAS can also identify cytoplasmic self-DNA leaked from the nucleus, contributing to autoimmune responses and inflammatory processes. STING, a key component of the cGAS signaling pathway, has been scientifically linked to the regulation of glucose and lipid metabolism. In a high-fat diet (HFD)-induced rat model, STING gene deletion significantly improved insulin sensitivity and glucose tolerance, underscoring its pivotal role in the metabolic disorders associated with T2DM [24]. In the pathological state of T2DM, activation of the cGAS-STING signaling pathway exerts profound effects on the cardiovascular system.

### 3.1. The cGAS-STING Pathway and Its Involvement in Inflammatory Responses Mediated by Immune Cells in Diabetes

Diabetes is not only a metabolic disorder but also severely compromises immune function, resulting in deficiencies in critical immune responses. Chronic hyperglycemia promotes oxidative stress by accumulating advanced glycation end-products (AGEs) and reactive Oxygen Species (ROS), which drive low-grade chronic inflammation and weaken immune efficacy [25,26]. During the development of T2DM, the polarization state of macrophages plays a crucial role. Macrophages are highly plastic and can shift their functional state based on signals from the local microenvironment. Under physiological conditions, they typically exhibit two main polarization phenotypes: M1, which is pro-inflammatory, and M2, which is anti-inflammatory and reparative [27]. In the pathogenic condition of T2DM, there is frequently an elevation of M1 macrophages that secrete several pro-inflammatory cytokines, such as TNF-α and IL-1β. These cytokines not only fuel inflammatory responses but also exacerbate insulin resistance, thereby driving the progression of diabetes-related complications [28]. A HFD not only alters metabolism but also activates the cGAS-STING pathway in adipocytes and macrophages, triggering autophagy in adipocytes. Macrophages, as the dominant pro-inflammatory immune cells in obese adipose tissue, exacerbate metabolic dysfunction in obese mice via cGAS-STING pathway activation [29]. Furthermore, studies in obese patients with pulmonary inflammation reveal elevated levels of STING+/CD68+ macrophages, where STING signaling drives pro-inflammatory activation in these cells, exacerbating obesity-associated inflammation [30]. Moreover, Tai et al. demonstrated in macrophages exposed to high glucose that inhibition of aberrant STING activation impairs autophagic function, thereby accelerating macrophage senescence [31]. Recently, stem cell therapy has emerged as a promising approach for T2DM treatment [32]. Although direct evidence linking mesenchymal stem cells (MSCs) to the suppression of the cGAS-STING pathway in T2DM is limited, studies have shown that human umbilical cord mesenchymal stem cells (HMSCs) promote the polarization of macrophages from an M1 to an M2 state, thereby mitigating islet dysfunction in T2DM mouse models [33]. Additionally, MSCs derived from adipose tissue or umbilical cords have been documented to induce a shift in macrophages toward an anti-inflammatory M2 phenotype, leading to increased M2 macrophage presence in the liver, adipose, and muscle tissues [34]. Further research suggests that HMSCs, through M2 macrophage activation, can inhibit the cGAS-STING pathway, counteracting liver injury caused by acetaminophen overdose [35]. These findings highlight a potential link between MSCs, M2 macrophages, and the cGAS-STING pathway. In T2DM patients, immune T cells exhibit a pronounced inflammatory phenotype, with a significant predominance of CD4^+^ T cells secreting Interferon-γ (IFN-γ) compared to control subjects, indicating heightened inflammatory activity of CD4^+^ T cells in T2DM [36]. Although cGAS expression is low in CD4^+^ T cells [37], the absence of cGAS or STING does not seem to affect glycolysis and oxidative phosphorylation in these cells [37], indicating that cGAS may not mediate DNA-stimulated metabolic processes in CD4^+^ T cells. Interestingly, STING is expressed at higher levels in mouse CD4^+^ T cells than in other immune cells [38,39]. Recent research has further explored the connection between DsbA-L and T cells, particularly in relation to the cGAS-STING pathway [40]. It is reasonable to speculate that DsbA-L may act as a link between T cells and the cGAS-STING pathway. Research has shown that in HFD-fed mice, a deficiency in DsbA-L disrupts mitochondrial function in T cells. Furthermore, knocking down DsbA-L in specific T cells reduces the production of IFN-γ and the accumulation of Treg cells, which contributes to alleviating obesity in these mice. Given the effects of STING agonists in HFD-induced obese mice [41], it is plausible to hypothesize that DsbA-L may inhibit the cGAS-STING pathway in T cells, potentially suppressing the development and progression of diabetes. However, further experimental validation is necessary to confirm this hypothesis and elucidate the precise molecular mechanisms involved.

### 3.2. The cGAS-STING Pathway and Gut Microbiota Dysbiosis in T2DM

Recent studies have identified bacterial DNA accumulation in the islet β-cells of obese mice, potentially triggering inflammatory responses and impairing insulin secretion through cGAS-STING pathway activation [42]. Bacteria have also been detected in the bloodstream and adipose tissues of individuals with obesity and T2DM. Compared to healthy individuals, the gut microbiota composition and its metabolites differ significantly in these patients [43]. Specifically, genera such as *Ruminococcus*, *Fusobacterium*, and *Bacteroides* are positively correlated with the onset of T2DM, while *Bifidobacterium*, *Akkermansia*, *Prevotella*, *Roseburia* and *Faecalibacterium* are inversely associated with the development of the disease [44]. Expansion of Gram-negative bacteria containing lipopolysaccharides (LPS) in their outer membranes disrupts intestinal tight junctions, increasing gut epithelial permeability. This shift reduces beneficial short-chain fatty acids (SCFA)-producing bacteria, weakening gut barrier function and promoting low-grade systemic inflammation—a key factor in T2DM pathogenesis [45,46]. The gut microbiota significantly impacts host metabolism through metabolites including LPS, SCFA, bile acids (BAs), and trimethylamine N-oxide (TMAO) [47]. LPS, a principal component of the cell wall of Gram-negative bacteria, can trigger a phenotypic transition in macrophages from an anti-inflammatory to a pro-inflammatory state, which is critical for initiating and sustaining inflammatory responses [48]. In the context of obesity and T2DM, LPS exposure in adipocytes may contribute to cell death and the formation of crown-like structures in inflamed adipose tissue [49]. Elevated LPS levels in the gut have been shown to reduce the expression of tight junction proteins, such as zonula occludens-1 and occludin, thereby compromising the permeability and integrity of intestinal epithelial cells [50]. With increased intestinal permeability, LPS can more readily cross the gut barrier and enter the circulatory system, leading to systemic low-grade inflammation. Recent studies suggest that LPS can induce myocardial damage in mice, where the deletion of STING significantly ameliorated cardiac injury by inhibiting LPS-induced pyroptosis, inflammation, and apoptosis in cardiomyocytes [51]. Recent research has revealed the substantial influence of microbiome-derived SCFAs, including butyrate, propionate, and acetate, on glucose metabolism and glucose homeostasis [52]. Notably, *Akkermansia muciniphila*, a mucin-degrading bacterium, has demonstrated beneficial effects on glucose metabolism by producing SCFAs through the degradation of host glycemic substances, which also contributes to the maintenance of a healthy body weight [53]. Among these SCFAs, butyrate, a critical metabolite, has been demonstrated to alleviate chronic kidney disease by influencing NLRP3-mediated pyroptosis via the STING/NF-κB/p65 signaling axis [54]. These findings collectively underscore the pivotal role of SCFAs and the STING signaling pathway in metabolic regulation. Moreover, the impact of gut microbiota dysbiosis on peroxisome proliferator-activated receptors (PPARs) has garnered considerable attention. Peroxisome Proliferator-Activated Receptor Gamma (PPARγ), in particular, has a strong link with the gut microbiota and brown adipose tissue, and is essential for the activation of brown adipose tissue, thereby facilitating the reduction in fat accumulation [55]. Under diabetic conditions, the activation of Peroxisome Proliferator-Activated Receptor Alpha (PPARα) can attenuate the cGAS-STING signaling pathway, leading to reduced inflammation and insulin resistance [56]. Additionally, therapeutic interventions such as Danshensu Bingpian Zhi have been shown to improve gut microbiota imbalance, mitigate weight gain, and enhance insulin sensitivity in diabetic murine models by increasing beneficial bacteria like *Akkermansia muciniphila* and modulating PPARγ activity [57]. Garlic-derived exosome-like nanoparticles (GaELNs) have emerged as a promising therapeutic strategy for T2DM [58]. Research suggests that GaELNs can ameliorate T2DM by activating *Akkermansia muciniphila* in the gut. Specifically, treatment with GaELNs has been shown to elevate levels of Amuc-1100, P9, and phosphatidylcholine (PC) in outer membrane vesicles (OMVs). The increased levels of Amuc-1100 and P9 are associated with higher plasma concentrations of glucagon-like peptide-1 (GLP-1), a crucial intestinal hormone that promotes insulin secretion and inhibits glucagon release, thus aiding in the reduction in blood glucose levels [59]. PC enrichment inhibits cGAS-STING signaling by blocking cytosolic DNA sensing, while synergizing with GLP-1 receptor activation to upregulate insulin receptor substrate (IRS) expression [60]. Bile acids and their associated receptor, Takeda G-protein-coupled receptor 5 (TGR5), are recognized for their significant involvement in cardiovascular and metabolic disorders through their impact on metabolic regulation and cardiac protection [61,62]. The imbalance of the gut microbiota results in reduced bile acid activity and a decline in the production of free and secondary bile acids. This imbalance also diminishes the activity of the TGR5 receptor, a crucial component of the immune response in T2DM [63]. Although the exact interplay between BA and the cGAS-STING signaling pathway in T2DM has not been completely elucidated, existing research offers valuable perspectives. For instance, in a mouse model of cholestasis, the use of the STING inhibitor C-170 reduced BA expression levels [64], suggesting a link between inflammatory responses and alterations in BA levels. It has also been reported that the gut microbiome in T2DM patients produced imidazole propionate through an alternative histidine metabolism pathway. Reports indicated that in T2DM, the gut microbiota produced imidazole propionate via an alternative metabolic pathway from histidine. This metabolite entered the liver through the portal vein, activating p38γ and subsequently inducing p62 phosphorylation, which deactivated insulin signaling via the mTORC1 pathway [65]. Conversely, research suggested that obesity-associated signals might enhance TBK1 activity and mTOR phosphorylation, synergizing with insulin pathways to boost mTORC1 and mTORC2 signaling. This mechanism may alleviate insulin resistance and enhance glucose homeostasis in the context of diet-induced obesity [66]. It is implied that microbial byproducts may modulate the host’s metabolic and inflammatory profiles, navigating through complex signaling networks.

### 3.3. The cGAS-STING Pathway and Mitochondrial Damage in Diabetes

**Figure 2:**
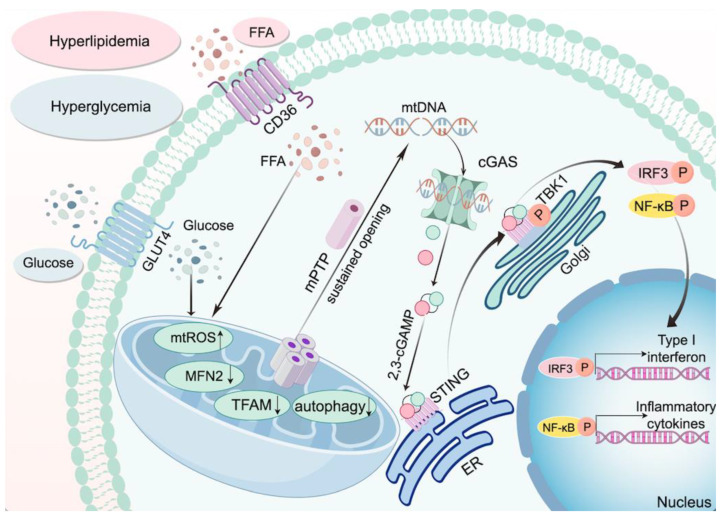
The cGAS-STING Pathway and Mitochondrial Damage in Diabetes. Hyperglycemia, hyperlipidemia, and insulin resistance lead to elevated levels of FFAs and mROS, which, in turn, impair mitochondrial function and disrupt cellular energy balance. Mitochondrial damage results in the release of mtDNA into the cytoplasm, and the released mtDNA activates the cGAS-STING pathway, triggering a cascade of inflammatory responses. This pathway involves the production of 2′,3′-cGAMP, a second messenger that binds to STING, thereby activating downstream signaling molecules such as TBK1 and IRF3. The activation of these molecules induces the transcription of pro-inflammatory cytokines and type I interferons, thereby aggravating myocardial injury.

Mitochondria, the central hubs of cellular energy production and stress integration, are efficiently cleared through dynamic quality control mechanisms—including fission, fusion, and mitophagy—under physiological conditions. However, in T2DM, mitochondria exhibit severe functional impairments, with oxidative DNA damage contributing to genomic instability [67]. In T2DM, mitochondrial dysfunction is primarily driven by the synergistic effects of hyperglycemia and free fatty acids (FFAs). Hyperglycemia increases mitochondrial reactive oxygen species (mtROS) production via GLUT4-mediated glucose uptake, while FFAs exacerbate mtROS accumulation through CD36 receptor internalization. Sustained oxidative stress induces persistent opening of the mitochondrial permeability transition pore (mPTP), facilitating mtDNA leakage into the cytoplasm. Additionally, hyperglycemia impairs mitochondrial integrity by suppressing the SIRT1/AMPK/PGC1α axis, triggering cardiomyocyte apoptosis and the release of mtDNA-containing extracellular vesicles, which can activate the cGAS-STING pathway upon uptake by adjacent fibroblasts [68,69,70,71]. PGC-1α, a key transcriptional coactivator, is critical for energy metabolism, mitochondrial biogenesis, and antioxidant stress. Studies show that PGC-1α expression is low in the islets of normal mice but markedly elevated in diabetic mice with compromised β-cell function [72]. In the placenta of gestational diabetes—also characterized by hyperglycemia—low PGC-1α and DsbA-L expression are thought to activate the cGAS-STING pathway [73]. Patients with T2DM exhibit elevated levels of glucose, fatty acids, and pro-inflammatory cytokines, along with reduced cardiac angiogenesis [74]. In DCM, elevated circulating fatty acids activate PPARα, promoting mitochondrial fatty acid uptake [75]. Downregulation of PPARα in monocytes from T2DM patients impairs mitochondrial function, shifts cellular metabolism toward glycolysis, and promotes mtDNA release, thereby activating the cGAS-STING pathway [56]. Xu et al. reported that diabetic mice with cardiomyocyte-specific TGR5 deletion exhibit increased cardiac fatty acid uptake and lipid accumulation due to enhanced CD36 palmitoylation and its translocation to the plasma membrane via palmitoyl acyltransferase DHHC4. Whereas activation of TGR5 prevents cardiac dysfunction and cardiolipotoxicity [61]. Notably, in diabetic conditions, TGR5 activation can disrupt the IP3R1-GRP75-VDAC1 axis that regulates Ca^2+^ transport from the endoplasmic reticulum to mitochondria. By preventing mitochondrial Ca^2+^ overload and subsequent mtDNA release, this intervention inhibits cGAS-STING activation and improves diabetic retinopathy [76]. Mitochondrial fusion protein 2 (MFN2) maintains mitochondrial dynamics, and its deficiency in diabetic MI/RI activates cGAS-STING-mediated myocardial injury. Conversely, MFN2 overexpression rescues PINK1/Parkin-dependent mitophagy [77]. Furthermore, in T2DM, both mitochondrial function and PINK1/Parkin-mediated mitophagy are impaired [78]. In PA-induced lipotoxic inflammation of adipocytes, PINK1 deficiency promotes mtDNA release, thereby activating the cGAS-STING pathway and driving inflammatory responses [79] Mitochondrial transcription factor A (TFAM) is essential for mtDNA transcription and replication. In cardiovascular and metabolic disorders, reduced TFAM levels are linked to mitochondrial stress that can cause mtDNA dysregulation. This dysregulation may cause mtDNA release, thereby triggering the cGAS-STING cascade and potentially worsening the condition [80,81]. Yuan et al. showed that FFA-induced mitochondrial damage causes mtDNA release, which subsequently triggers the cGAS-STING-IRF3 pathway. Within this pathway, phosphorylated IRF3 interacts with the Hippo pathway, initiated by Mammalian Ste20-like kinases 1, further inhibiting cell viability, proliferation, and angiogenic processes [82]. It has been shown that PA-induced ROS can oxidatively damage mtDNA in mitochondria, causing its leakage, which activates the cGAS-STING pathway, triggers NLRP3-mediated pyroptosis, and ultimately leads to myocardial hypertrophy [83]. Similarly, Xu et al. demonstrated that increased ROS levels cause mtDNA leakage and cGAS-STING activation, which in turn triggers downstream NF-κB and IRF3 signaling that promotes IL-18 and IL-1β expression in myocardial cells—thereby exacerbating mitochondrial damage and further mtDNA release [84]. Lin et al. found a significant decrease in FNDC5/Irisin expression in db/db mice; in these mice, FNDC5/Irisin alleviates DCM by activating the integrin αV/β5-AKT pathway, which is key to reducing mitochondrial damage. Moreover, FNDC5/Irisin mitigates oxidative and nitrosative stress, further improving DCM conditions [85]. Another study found that irisin enhances mitochondrial function by activating the MITOL signal, modulates GSDMD-mediated pyroptosis, and reduces cGAS-STING expression, thereby improving cardiac remodeling in DCM [86]. Leptin, a hormone secreted by adipose tissue, regulates mitochondrial function both indirectly through intracellular signaling pathways and directly by targeting mitochondria. It participates in energy metabolism regulation, enhances proton leakage, promotes the opening of the mitochondrial permeability transition pore, and induces cardiomyocyte apoptosis, thereby impacting mitochondrial homeostasis and cardiac remodeling [87]. Endogenous leptin exerts pro-hypertrophic effects on the heart, whereas exogenous leptin has been shown to markedly attenuate cardiac hypertrophy and fibrosis by modulating the cGAS-STING signaling pathway and Opa1-mediated mitochondrial fusion [88,89]. DsbA-L is a versatile protein whose functions extend beyond self-polymerization. It plays a pivotal role in regulating energy homeostasis and enhancing energy expenditure in mice, making a significant contribution to obesity mitigation. Research shows that DsbA-L is abundantly expressed in adipose tissue and that its levels are inversely correlated with obesity prevalence in both mice and humans [90]. In the white adipose tissue of obese mice, cGAS-STING is overactivated while DsbA-L levels are low. In contrast, mice with fat-specific DsbA-L overexpression are protected from obesity-induced inflammation and insulin resistance by inhibiting the cGAS-STING pathway [41]. In HFD-induced obese diabetic mice, TBK1 activation—a kinase downstream of STING—negatively impacts AMPK, a key regulator of energy homeostasis, by directly inhibiting AMPKα phosphorylation at Thr172. This inhibition disrupts mitochondrial ATP metabolism and energy homeostasis [91]. Notably, STING activation suppresses AMPK expression, and conversely, AMPK knockout further activates the cGAS-STING-mediated inflammatory response, exacerbating high-fat diet–induced cardiac abnormalities [92]. IQGAP1, a GTPase-activating protein with IQ motifs, acts as a scaffold in multiple signal transduction pathways by interacting with numerous partners to regulate diverse biological processes. Specifically, IQGAP1 binds to the α1 subunit of AMPK and directly interacts with CaMKK2, which is crucial for full AMPK activation [93]. Consequently, IQGAP1 not only promotes AMPK signal transduction but also may play a key role in regulating the cGAS-STING pathway. Cheng et al. demonstrated that silencing IQGAP1 via adeno-associated virus (AAV) in ApoE^−^/^−^ and Ldlr^−^/^−^ C57BL/6J mice on a high-fat diet alleviates atherosclerosis by suppressing mtDNA-cGAS-STING–mediated endothelial pyroptosis [94]. Figure 2 illustrates how mitochondrial damage under diabetic conditions contributes to the activation of the cGAS–STING pathway and subsequent inflammatory responses.

### 3.4. cGAS-STING and Epigenetic Modifier Changes in Diabetes

Long non-coding RNAs (lncRNAs), defined as RNA molecules longer than 200 nucleotides that do not encode proteins, are key regulators of gene expression at the epigenetic, transcriptional, and post-transcriptional levels [95]. In T2DM and its complications, lncRNAs play critical roles, particularly in the pathogenesis of DCM [96]. For instance, Qi et al. found that in DCM patients, downregulation of myocardial lncRNA HOTAIR reduces cardiomyocyte viability and worsens DCM progression, whereas its upregulation activates the PI3K/Akt pathway, improving DCM. Meanwhile, in diabetic rats, downregulation of myocardial lncRNA H19 decreases miR-675, leading to increased cardiomyocyte apoptosis [97,98]. In 2021, lncRNA ZNF593-AS was identified as a novel regulator of contractile dysfunction in DCM [99]. Further Xie et al. showed that upregulating lncRNA ZNF593-AS in T2DM mouse models can mitigate cardiac dysfunction. This occurs as ZNF593-AS inhibits IRF3 phosphorylation, a downstream component of the STING pathway, thereby preventing its nuclear translocation and the release of pro-inflammatory factors [100]. MicroRNAs (miRNAs) are small non-coding RNAs (~22–23 nucleotides) that regulate gene expression post-transcriptionally by binding to complementary sequences in the 3′ untranslated regions (3′UTRs) of target mRNAs. They are crucial regulators of the cGAS-STING signaling pathway, which is vital for immune responses. The 3′UTRs of cGAS and STING mRNAs have potential binding sites for various miRNAs [101,102]. For example, miR-23a/b directly binds to the 3′UTR of cGAS mRNA, inhibiting its expression and suppressing cGAS-mediated innate immune responses [103]. Similarly, miR-210, miR-24, and miR-24-3p target the 3′UTR of STING mRNA to downregulate its expression and inhibit the STING signaling pathway [104]. Moreover, miRNAs may indirectly inhibit the cGAS-STING pathway by suppressing mitochondrial fission and apoptosis, providing a protective effect on the heart [105]. Specific miRNAs, such as hsa-miR-611, hsa-miR-5192, and hsa-miR-1976, may regulate the STING pathway by targeting TMEM173 and CHUK mRNAs. The expression levels of these miRNAs progressively increase from healthy individuals to those with prediabetes and T2DM, particularly in T2DM patients, suggesting their potential involvement in diabetes development [106].

### 3.5. cGAS-STING and Cell Death in Diabetes

In individuals with T2DM, cellular demise is a complex process mediated by diverse mechanisms, including apoptosis, necrosis, pyroptosis, ferroptosis, autophagy, cuproptosis, and pan-cellular death. Each pathway plays a distinct role in physiological and pathological contexts [107]. Apoptosis is a tightly regulated mechanism that removes excess or damaged cells, preserving tissue homeostasis. Marked by unique structural changes like cell shrinkage, chromatin condensation, and apoptotic body formation, it allows phagocytes to clear cells without inflammation. In T2DM, these cell death mechanisms are often disrupted. A major factor in disease progression is impaired insulin secretion, largely due to β-cell apoptosis. High glucose metabolism in β-cells can cause double-strand DNA breaks and activate the tumor suppressor protein p53, leading to β-cell dysfunction and failure [108]. The co-occurrence of hyperglycemia and hyperlipidemia in T2DM further exacerbates β-cell apoptosis [109]. Necroptosis is a regulated form of programmed cell death involving the BCL-2 family protein PUMA. PUMA is transcriptionally induced in a RIP3/MLKL-dependent manner and promotes mtDNA release into the cytoplasm, which subsequently activates DAI/Zbp1 and STING [110]. In diabetic retinopathy, STING activation triggers the RIP1/RIP3-MLKL signaling pathway, leading to cell membrane rupture, leakage of intracellular contents, and subsequent inflammatory responses, ultimately promoting pathological angiogenesis [111]. Interestingly a similar mechanism is observed in DCM, where STING activation via the RIP1/RIP3-MLKL pathway induces necroptosis and inflammation in cardiomyocytes, contributing to myocardial dysfunction [112]. Pyroptosis, an inflammatory lytic cell death is closely associated with the activation of inflammation. Pathological stimuli such as lipotoxicity trigger NLRP3 inflammasome assembly, converting pro-caspase-1 into its active form. Activated caspase-1 catalyzes dual pro-inflammatory actions: maturation of IL-1β and IL-18, and cleavage of GSDMD into its pore-forming N-terminal fragment (GSDMD-N). These pores facilitate the release of cytoplasmic contents, perpetuating sterile inflammation [113,114]. In DCM, mtDNA release activates the cGAS-STING pathway, which promotes IRF3 activation and, in turn, triggers NLRP3 inflammasome activation and increases GSDMD expression in cardiomyocytes. Notably, aberrant GSDMD expression in the hearts of STING-deficient T2DM mice is reversed, underscoring the crucial role of STING in DCM-related pyroptosis [83]. Ferroptosis, a novel type of cellular demise, is defined by the buildup of intracellular iron and the ROS, especially when the antioxidant capacity of cell is diminished. Ferroptosis is intricately associated with various diseases, including metabolic and cardiovascular conditions [115]. Recent studies have shown that in HFD-fed rat models and PA-induced lipotoxicity cell models, activation of the cGAS-STING pathway mediates ferroptosis in cardiomyocytes. While the HFD-fed rats treated ferroptosis inhibitors showed significant improvements in cardiac structure and function [116]. Copper-induced cell death, known as cuproptosis, is an emerging mechanism initiated by copper ionophores such as Disulfiram, Elesclomol, and NSC319726 [117]. In C8-D1A cells, it promotes the release of mitochondrial mtDNA and activates the cGAS-STING pathway, triggering pyroptosis [118]. In DCM, dysregulated copper metabolism elevates serum copper levels while reducing hepatic copper clearance, potentially driving cuproptosis [119]. The associated mtDNA release and cGAS-STING activation contribute to mitochondrial damage, inflammation, and cell death, suggesting a critical role for cuproptosis in DCM pathogenesis. Thus, the cGAS-STING pathway is intricately involved in multiple modes of cell death in diabetes, contributing to a complex interplay of inflammation, cell death, and metabolic dysregulation. Targeting this pathway may offer novel therapeutic strategies to protect against cell death and ameliorate diabetic complications.

### 3.6. cGAS-STING and Other Factors in T2DM

For patients with T2DM, it is essential to not only tightly regulate blood glucose and lipid levels but also vigilantly monitor for potential hypoglycemia, particularly due to excessive insulin use, as it may precipitate cardiovascular events. Research indicates that while hypoglycemic conditions marginally increase the expression of cGAS and STING, their direct involvement in pyroptosis remains limited [120]. Exosomes, small extracellular vesicles secreted by cells, play a pivotal role in the immune response and metabolic disorders associated with T2DM. By participating in intercellular communication and immune regulation, exosomes influence the function of immune cells and the production of cytokines [121,122,123]. Obesity can lead to a reduction in CRIg^+^ macrophages in the liver, which are essential for clearing microbial DNA and microvesicles (mEVs) from the bloodstream. When intestinal barrier function is compromised, an obese host may experience an increased influx of microbial DNA and exosomes into the bloodstream, impacting metabolic tissues. If the gut microbial DNA carried by these exosomes activates the cGAS-STING pathway, it could trigger an inflammatory response, impair insulin signaling, and promote the development of insulin resistance [124]. Recent research has shown that GaELN can induce the release of outer membrane vesicles (OMVs) from *Akkermansia muciniphila*, leading to the suppression of inflammatory cytokine expression in the brains of HFD-induced mice. GaELN treatment enhances the expression of PC in OMVs, inhibiting the activation of cGAS-STING in microglia, which offers a new therapeutic strategy for reversing T2DM [60]. Furthermore, it is well-established that aerobic exercise can enhance insulin sensitivity in patients with T2DM and effectively reduce blood glucose, blood pressure, and lipid levels [125]. Research has also demonstrated that aerobic exercise significantly ameliorates cardiac function in diabetic mice by suppressing the cGAS-STING pathway [126]. Based on these findings, we recommend that individuals with T2DM engage in aerobic exercise to improve glycemic and lipid control.

## 4. cGAS-STING and Cardiovascular Complications in Diabetes

**Figure 3:**
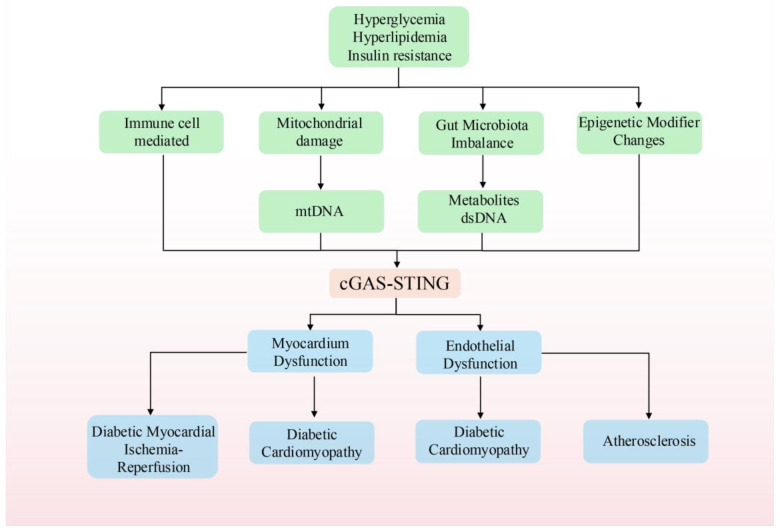
Activation of the cGAS-STING signaling pathway in T2DM. Hyperglycemia, hyperlipidemia, and insulin resistance are hallmark metabolic features of diabetes and its complications. These metabolic abnormalities can induce mitochondrial dysfunction and gut microbiota dysbiosis through multiple mechanisms, leading to the release of mtDNA and bacterial-derived DNA into the cytoplasm. These exogenous or damage-associated DNA fragments act as pathological signals that activate the cytosolic cGAS-STING signaling pathway. The activation of this pathway not only drives immune responses but is also modulated by epigenetic modifications, further amplifying the inflammatory cascade. The persistent activation of this inflammatory signaling contributes to two major cardiovascular pathological changes. On one hand, it leads to myocardial dysfunction, including DCM and diabetes-related MI/RI. On the other hand, endothelial dysfunction progressively develops, promoting the onset and progression of atherosclerosis.

The cGAS-STING signaling pathway plays a critical role in mediating cardiovascular complications associated with diabetes, particularly in DCM, MI/RI, and vascular endothelial injury. In the diabetic milieu, chronic hyperglycemia and hyperlipidemia promote the release of mtDNA into the cytoplasm—a process further exacerbated by cell death—which activates the cGAS-STING pathway. This activation triggers inflammatory responses and fibrosis, ultimately contributing to the structural and functional deterioration of the heart and vascular damage. The underlying mechanism is illustrated in Figure 3.

### 4.1. cGAS-STING and Diabetic Cardiomyopathy

DCM is a distinct heart disease associated with diabetes, primarily characterized by heart failure. Even when other cardiovascular conditions, such as hypertension and coronary artery disease, are excluded, patients with DCM still exhibit significant myocardial structural changes and persistent abnormalities in ventricular systolic and diastolic function [127,128]. Research by our team has demonstrated a lipotoxicity-driven pathological cascade in cardiomyocytes, wherein PA-induced mitochondrial dysfunction precipitates DCM progression. Our study demonstrated that PA overload disrupts mitochondrial homeostasis by inducing mtDNA leakage and cGAS-STING pathway hyperactivation, ultimately leading to NLRP3 inflammasome-mediated cardiomyocyte pyroptosis. Pharmacological intervention with the cGAS inhibitor RU.521 or STING antagonist C176-1 effectively attenuated this myocardial injury [83]. Further studies by Ma et al. corroborate these findings, showing similar results that suppressing the expression of STING can block the inflammatory and apoptotic responses in obesity-related diabetes mouse model [84]. Emerging evidence has highlighted Meteorin-like (Metrnl), a neurotrophic factor homolog secretory peptide, as a promising cardioprotective agent. Pioneering research by Lu et al. demonstrated that Metrnl administration effectively ameliorates impaired autophagy in DCM murine models through dual molecular mechanisms. The therapeutic peptide orchestrates myocardial homeostasis by sequentially activating the LKB1/AMPK/ULK1 signaling axis and subsequently inducing STING dephosphorylation. This coordinated action facilitates mitochondrial repositioning and promotes the formation of a STING-TRAF2 (TNF receptor-associated factor 2) complex, which undergoes selective ubiquitin-mediated proteasomal degradation. The resultant autophagic flux enhancement significantly improves cardiac systolic function and metabolic adaptation in diabetic myocardium [129]. Additionally, studies have highlighted the cardioprotective effects of Irisin in DCM induced by T2DM, primarily through the reduction in oxidative stress and inflammatory responses [85,130]. Recent investigations by Lu et al. have revealed that Irisin upregulated mitochondrial ubiquitin ligase MITOL/MARCH5, which orchestrated the suppression of STING-dependent pro-inflammatory cascades and attenuated GSDMD-executed pyroptotic cell death [86]. This concerted action effectively mitigates maladaptive cardiac structural remodeling in DCM models. IL-37 emerges as a critical endogenous modulator of mitochondrial resilience, sustaining the activity of the SIRT1-AMPK-PGC1α metabolic sensor axis. Through this pathway, IL-37 preserves mitochondrial homeostasis, curbs the extracellular release of mtDNA-containing vesicles, and decelerates DCM progression. The synergistic interplay between Irisin-mediated STING inhibition and IL-37-driven mitochondrial preservation delineates a promising therapeutic paradigm for targeting metabolic-inflammatory crosstalk in diabetic cardiac pathology [71]. Recent research by Chen et al. discovered that in DCM mouse models, the expression of BRG1, a key factor in DNA repair, is reduced. The loss of BRG1 impairs the repair of DNA double-strand breaks (DSBs), leading to the accumulation of dsDNA in the cytoplasm. This accumulation activates the cGAS-STING pathway, triggering inflammatory responses and apoptosis, which further contributes to the progression of DCM [131,132]. Collectively, these findings underscore the cGAS-STING signaling pathway as a critical regulator of immune-inflammatory responses in DCM, emphasizing its potential as a therapeutic target for alleviating complications associated with diabetes.

### 4.2. cGAS-STING and Diabetic Myocardial Ischemia/Reperfusion Injury

In patients with T2DM, myocardial I/R injury is notably more severe due to the abnormal cellular stress responses induced by chronic hyperglycemia and glucose fluctuations. These abnormal stress responses contribute to exacerbated cardiac injury during myocardial I/R injury events. In diabetic mouse models, the expression of mitochondrial fusion protein MFN2 was significantly reduced. This defect exacerbates mitochondrial damage and causes mtDNA leakage, thereby activating the cGAS-STING signaling pathway and aggravating cardiac injury [77]. Conversely, MFN2 overexpression markedly reduced mtDNA leakage and indirectly suppressed pathway activation. In addition to MFN2, research has also highlighted the role of AlkB homolog 5 (ALKBH5) and stress granules (SGs) in the context of diabetic myocardial I/R injury. Under diabetic myocardial I/R conditions, the expression levels of ALKBH5 and SGs are significantly reduced, contributing to increased myocardial tissue damage, cardiomyocyte apoptosis, and larger infarct sizes. However, overexpression of ALKBH5 using adeno-associated virus vectors has been shown to significantly mitigate these effects. ALKBH5 overexpression leads to a reduction in the expression levels of cGAS and STING, as well as a decrease in inflammatory cytokines such as IL-1β and TNF-α. These findings suggest that ALKBH5 may alleviate diabetic myocardial I/R injury by promoting the expression of SGs and inhibiting the activation of the cGAS-STING pathway [133].

### 4.3. cGAS-STING and Diabetic Atherosclerosis

Atherosclerosis is a common complication in diabetic patients, often driven by aortic endothelial injury, which is closely associated with hyperglycemia-induced vascular dysfunction. Under hyperglycemic conditions, endothelial cells are particularly vulnerable to damage, leading to vascular endothelial dysfunction, a key contributor to the progression of atherosclerosis [134,135]. Research conducted by An et al. demonstrated that in a T2DM mouse model, elevated blood glucose levels could activate the STING signaling pathway, resulting in diabetic aortic endothelial injury. Further in vitro studies revealed that inhibiting the STING signaling pathway in endothelial cells effectively blocked the activation of the IRF3/NF-κB pathway induced by high glucose levels [136]. Mao et al. explored the impact of obesity-induced FFAs on endothelial cells in an obese mouse model. Their findings indicated that FFAs caused mitochondrial dysfunction, leading to the activation of the STING-IRF3 signaling pathway. This activation results in the upregulation of adhesion molecules, including vascular cell adhesion molecule 1 (VCAM-1) and intercellular adhesion molecule 1 (ICAM-1), in endothelial cells. These molecules increase the adhesion of monocytes to endothelial cells, which in turn exacerbates endothelial inflammation and promotes the progression of atherosclerosis [137]. Moreover, a study revealed that in the adipocytes of T2DM mice, the expression level of IQGAP1, a protein involved in cytoskeletal organization and cellular signaling, was significantly lower than in normal control groups [138]. Reduced IQGAP1 expression in endothelial cells, particularly when induced by PA, was found to exacerbate mitochondrial damage, activate the mtDNA sensor cGAS-STING signaling pathway, and ultimately lead to endothelial cell pyroptosis. This process accelerates the progression of atherosclerosis in diabetic conditions [94].

## 5. Therapeutic Potential of cGAS-STING in T2DM and Its Cardiovascular Complications

Preclinical studies have shed light on the role of immune-inflammatory responses mediated by the cGAS–STING pathway in the progression of T2DM. In this review, we comprehensively examine the immune-inflammatory mechanisms associated with the cGAS–STING signaling pathway and explore a range of potential therapeutic strategies for T2DM and its cardiovascular complications. This includes cGAS inhibitors, STING inhibitors, and TBK1 inhibitors, as well as degraders targeting the cGAS–STING signaling pathway. In addition, we summarize recent advances in the cardioprotective effects of herbal formulas and natural compounds that exert regulatory actions via the cGAS–STING pathway. Our goal is to provide a comprehensive knowledge framework linking the cGAS–STING pathway to T2DM and its cardiovascular sequelae, offering valuable guidance for future research and clinical applications.

### 5.1. cGAS Inhibitors

cGAS, acting as a DNA sensor and key enzyme, can be modulated by small molecule inhibitors through three main mechanisms: (1) Inhibitors of cGAS acetylation modification; (2) Inhibitors that block the binding of DNA to cGAS; (3) Directly targeting the catalytic site of cGAS to disrupt cGAMP production [139]. The first category includes aspirin, which, in addition to its clinical use as an antiplatelet agent for antithrombotic therapy, can effectively inhibit cGAS activity by enhancing its acetylation at Lys 384/394/414 [140]. The second category comprises drugs like A151, hydroxychloroquine (HCQ), suramin, quinacrine (QC), and X6, all of which competitively bind to cGAS against dsDNA [141,142]. Additionally, suramin can effectively inhibit cGAS, reduce the migration and proliferation of vascular smooth muscle cell, decrease neointimal hyperplasia, and protect blood vessels from further damage [143,144]. The third category includes RU.521, G150, G108, and PF-06928215, which interfere with cGAMP production by competitively binding with ATP or GTP substrates and the cGAMP product of cGAS [145,146]. For instance, RU.521 can effectively inhibit cGAMP synthesis induced by PA in DCM cells, blocking cGAS expression and subsequent STING activation, consistent with previous findings in septic mouse models [147]. Moreover, the substrate competitive inhibitor PF-06928215 stands out for its high affinity to cGAS, showing an IC50 value of 4.9 μM and a Kd affinity of 0.2μM [83,148]. Research results indicate that PF-06928215 has significant inhibitory effects on cGAS activity in both in vitro and in vivo experiments. Interestingly, PF-06928215 has not yet shown activity in cellular cGAS assays [139]. However, recent research progress shows that it can effectively alleviate the contractile dysfunction of cardiomyocytes induced by PA [92]. Additionally, tetrahydro-γ-carboline derivatives (compound 25) interact with the catalytic site of cGAS to produce anti-inflammatory effects, showing strong inhibition of human and murine cGAS at cellular levels with IC50 values of 1.38μM and 11.4μM, respectively. In in vivo experiments using LPS-induced mouse inflammation models, compound 25 was administered intraperitoneally at a dose of 30 mg/kg, significantly reducing the production of pro-inflammatory cytokines, proving to be a promising cGAS inhibitor [149]. Notably, the first cGAS inhibitor to enter clinical development, VENT-03, was developed by Ventus Therapeutics using its proprietary structural biology and computational chemistry platform ReSOLVE™, enabling it to target cGAS with high affinity by disrupting cGAMP production, and it also has the advantage of oral administration [150].

### 5.2. STING Inhibitors

STING, a critical downstream effector in the cGAS signaling pathway, plays a pivotal role in the regulation of immune-inflammatory responses. Due to STING’s significant involvement in various pathological processes, researchers have developed several STING inhibitors, utilizing insights from its crystal structure and regulatory mechanisms. These inhibitors can be broadly classified into four categories: (1) inhibitors targeting the CDN binding pocket of STING, (2) inhibitors that prevent STING palmitoylation, (3) competitive inhibitors that bind to the cGAMP binding site and, (4) directly binds to the active site of STING [151]. The first category, exemplified by Astin C, includes natural cyclopeptides derived from the traditional Chinese medicine Aster tataricus. Astin C exhibits potent biological activity by inhibiting the cGAS-STING signaling pathway. Specifically, it binds to the C-terminal activation pocket of STING, thereby preventing the recruitment of IRF3 and preserving the functional interaction between STING and TBK1. This interaction is crucial in reducing auto-inflammatory responses, as demonstrated in Trex1-deficient mouse models and macrophages [152]. Furthermore, Astin C has shown potential in mitigating PA-induced myocardial cell damage, suggesting its promise as a therapeutic agent for DCM [92]. Another promising inhibitor, SN-011, has been shown through computer-aided simulations to bind the CDN pocket of STING with greater affinity than cGAMP. This binding stabilizes STING in an inactive state, effectively inhibiting the production of IFN-I and pro-inflammatory cytokines. SN-011 has exhibited good tolerance in Trex1 gene knockout mouse models, positioning it as a potential lead compound for treating diseases associated with STING dysregulation [153]. Gelsevirine, an alkaloid extracted from the traditional Chinese herb Gelsemium elegans Benth, also targets the CDN binding pocket of STING with high affinity. By competitively inhibiting STING activation, Gelsevirine not only prevents its pathological activation but also promotes K48-linked ubiquitination of the STING protein. This process facilitates STING degradation via recruitment of TRIM21, offering another potential therapeutic strategy for diseases linked to STING overactivation [154]. The second class of STING inhibitors, represented by nitrofuran derivatives, includes C-176, H-151, and C-178. These compounds specifically target Cys91 on the STING protein, blocking the palmitoylation process necessary for its activation [151,155]. Studies have shown that C-176 and H-151 have significant effects in preventing cardiomyopathy and improving remodeling after MI, making them suitable for preclinical studies of MI, myocardial I/R injury accompanied by T2DM, and DCM [155]. Notably, C-176 has also been confirmed to inhibit the dysfunction and apoptosis of endothelial cells caused by STING under high glucose conditions [77,136]. H-151 disrupts the palmitoylation of STING, preventing the formation of STING polymer complexes, reducing the expansion of infarct areas and scar formation, significantly restoring left ventricular systolic function in diabetic ischemia–reperfusion mouse models, and reducing the size of MI [77]. Additionally, nitro fatty acids (NO2-FAs) are special compounds formed by the reaction of nitric oxide (NO) with unsaturated fatty acids. They have similar effects to nitrofuran derivatives, being able to inhibit the STING signaling pathway. The inhibition of this pathway is particularly important during viral infections because nitro fatty acids can nitrosylate the palmitoylation site of STING, thereby inhibiting its activity. This process has been found to have a protective effect on cardiovascular health [156]. CXA-10, a nitro fatty acid with a strong safety profile, is currently undergoing Phase II clinical trials for pulmonary arterial hypertension (NCT04125745, NCT04053543, NCT03449524) [157]. The third type, exemplified by tetrahydroisoquinoline acetic acid (compound 18), can effectively stabilize the inactive state of the STING protein. It binds to the cGAMP binding site in a 2:1 ratio, replacing the original cGAMP. It binds tightly to specific amino acid residues Thr263 and Thr267 of the STING protein, has a slow dissociation rate, and good oral bioavailability. In vitro experiments have shown that tetrahydroisoquinoline acetic acid compound 18 can significantly inhibit cGAMP-dependent signaling [158]. Finally, the fourth class of STING inhibitors, represented by the cyclin-dependent kinase (CDK) inhibitor palbociclib, functions by targeting the Y167 residue of STING to block its dimerization and translocation [159].

### 5.3. TBK1 Inhibitors

TBK1 is a pivotal downstream kinase in the STING signaling pathway, essential for propagating the STING-mediated immune response. Amlexanox, an FDA-approved drug with notable anti-inflammatory properties, is primarily utilized for the treatment of asthma and recurrent aphthous ulcers [160]. As a small molecule inhibitor, Amlexanox exhibits a high affinity for TBK1, effectively blocking the phosphorylation of STING at Ser366—a modification induced by TBK1. Recent studies have also highlighted its potential therapeutic benefits in addressing obesity, MI, and other related conditions [161,162,163]. TBK1 inhibitors act by competitively occupying its ATP-binding pocket, thereby preventing ATP access and abrogating kinase activity [164]. Notably, BX795 and MRT67307—both featuring a 2,4-diaminopyrimidine core common to many ATP-competitive kinase inhibitors—preferentially bind the phosphorylated (active) form of TBK1 [165,166]. Crystal structures reveal their aromatic rings mimic the adenine moiety of ATP, with BX795 making additional contacts near the triphosphate region and SU6668 engaging the opposite side of the phosphate-binding site [167]. Similarly, GSK8612, another 2,4-diaminopyrimidine derivative, is predicted (via docking into the TBK1–MRT67307 complex) to hinge on Cys89 through its N1 nitrogen and adjacent NH linker, while its sulfonamide NH_2_ forms hydrogen bonds with Asn140 and Asp157—interactions that underlie its high affinity [168]. GSK8612 exhibits superior selectivity for TBK1 over related kinases IKKε and AAK1, although its lower affinity for phosphorylated TBK1 suggests that its inhibitory potency may vary with TBK1’s activation state. Collectively, TBK1 inhibitors have shown protective effects against cardiovascular and metabolic diseases, offering a promising strategy for future treatments. These studies have developed multiple classes of small-molecule inhibitors targeting the cGAS–STING–TBK1 signaling pathway, and their representative compounds and mechanisms of action are summarized in Table 1.

### 5.4. cGAS/STING Degraders

In addition to inhibitors targeting the cGAS/STING pathway, cGAS/STING degraders represent another class of molecules playing a significant role in immune regulation [169]. For cGAS degradation, studies show that K48-linked ubiquitination acts as a recognition signal for p62-dependent selective autophagy, effectively promoting cGAS breakdown [170]. Furthermore, the cullin-RING ubiquitin ligase 5 (CRL5) complex has been confirmed to specifically target nuclear cGAS and mediate its degradation [171]. Notably, a novel degrader, TH35, developed using Proteolysis-Targeting Chimeras (PROTACs) technology, achieves efficient and selective cGAS degradation by recruiting cGAS via CRBN. TH35 significantly suppresses dsDNA-induced cGAS pathway activation and exhibits low cytotoxicity in both human and mouse cells [172]. 2′3′-cGAMP phosphodiesterases are enzymes that specifically degrade 2′3′-cGAMP, the crucial second messenger in the cGAS-STING signaling pathway, thereby effectively inhibiting pathway activation [173]. ENPP1, the first identified 2′3′-cGAMP degrader, belongs to the ENPP family and contains a phosphodiesterase domain capable of hydrolyzing nucleotides. By specifically degrading 2′3′-cGAMP, ENPP1 effectively inhibits cGAS-STING pathway activation, potentially influencing the host’s immune response to tumors [174]. Expanding on this, Zhang’s team further discovered that SMPDL3A, an enzyme induced by the liver X receptor-mediated lipid metabolic pathway, can also selectively degrade 2′3′-cGAMP within the cGAS-STING pathway, thereby suppressing the activation of innate immune responses [175]. For STING degradation, E3 ubiquitin ligases such as RNF5, TRIM30a, and TRIM29 can mediate K48-linked ubiquitination, promoting STING degradation via the proteasomal pathway [176,177,178]. Additionally, STING can be degraded through an endosomal sorting complex required for transport (ESCRT)-dependent microautophagy mechanism, where K63-linked ubiquitination at Lys288 is crucial for preventing STING overactivation [179]. The endoplasmic reticulum-associated ubiquitin ligase HRD1 also participates in regulating the homeostasis of nascent STING protein [180]. On the other hand, the PROTAC-based STING degrader UNC9036 (DC_50_ = 227 nM) achieves selective STING degradation through an elegant mechanism: its diABZI fragment first binds to and activates STING, inducing its phosphorylation. Subsequently, UNC9036’s VHL ligand domain recruits the VHL E3 ligase, targeting the phosphorylated STING and leading to its proteasome-dependent degradation [181]. Beyond cGAS and STING, researchers have also developed degraders for their downstream kinase, TBK1. Crew and colleagues designed a series of TBK1-targeting PROTACs, with the representative compound “3i” demonstrating excellent degradation performance in in vitro experiments: DC_50_ of approximately 12 nM and a maximum degradation rate (Dmax) of 96%. It also showed over 50-fold selectivity for TBK1 compared to its homologous kinase IKKε, effectively inducing TBK1 degradation [182]. Furthermore, Guo et al. recently reported a series of TBK1 degraders, including PROTACs and molecular glues. Among these, “degrader 30” was identified as the most effective molecular glue degrader, promoting TBK1 degradation in cells in a dose- and time-dependent manner. Mechanistic studies revealed that degrader 30 recruits the E3 ubiquitin ligase RNF126 to direct TBK1 to the ubiquitin-proteasome pathway for degradation [183]. Although research has yet to clearly define the role of cGAS/STING degraders in diabetes and its associated cardiovascular complications, their established function within the cGAS-STING signaling pathway suggests significant potential. The development of these degraders as therapeutic agents holds immense promise for future clinical applications. To provide a clearer illustration of these findings, representative cGAS/STING degraders and their molecular mechanisms are summarized in Table 2.

### 5.5. Herbal Medicines and Monomers

Traditional Chinese medicine (TCM) has a history of several thousand years in clinical practice in China, and its potential in mitigating cardiac damage and immune inflammation has been widely acknowledged. Modern pharmacological studies indicate that TCM not only exhibits significant efficacy but, in some cases, even surpasses traditional therapies. This advantage is primarily attributed to the diverse bioactive phytochemicals in TCM and their multi-target mechanisms of action. Additionally, TCM has demonstrated a relatively lower incidence of side effects in clinical applications [184]. For patients with T2DM complicated by cardiovascular conditions, the adjunctive use of TCM can be personalized according to specific pathophysiological characteristics. Recent studies have shown that Mailuoning oral liquid significantly improves the thrombosis and gangrene formation in the thromboangiitis obliterans (TAO) rat model. The underlying mechanism of action is closely related to the inhibition of the cGAS-STING-IRF3 signaling pathway [185]. Among commonly used medicinal herbs, *Astragalus membranaceus* and *Salvia miltiorrhiza* have garnered considerable attention for their pharmacological effects. The active compounds Astragaloside IV (As-IV) and Tanshinone IIA (Ta-IIA) demonstrate significant cardiovascular protective properties [186,187]. Studies have shown that the combination of As-IV and Ta-IIA (Co) exhibits synergistic effects across multiple aspects: compared to individual compounds, Co more effectively reduces MI area, lowers serum myocardial enzyme levels, and promotes recovery of myocardial contractile function. Additionally, Co demonstrates stronger biological effects in terms of anti-apoptosis, antioxidant stress, and anti-inflammatory actions. Molecular mechanism studies further confirm that Co exerts myocardial protection by enhancing the inhibition of STING protein phosphorylation and its downstream signaling pathways [188]. Tetrandrine, a bisbenzylisoquinoline alkaloid extracted from the traditional Chinese medicine *Stephania tetrandra*, has been shown to exhibit significant anti-inflammatory and immunomodulatory properties [189]. In the pathological progression of atherosclerosis, macrophage inflammation activation is a critical pathogenic mechanism. Studies have demonstrated that tetrandrine specifically inhibits the STING-TBK1 signaling pathway, significantly reducing macrophage inflammatory activation. This action is crucial in decelerating the development and advancement of atherosclerotic plaques [190]. Ginsenoside Rb1, one of the major active ingredients extracted from *Panax ginseng*, has been extensively validated for its multifaceted cardiovascular protective effects [191]. At the molecular level, studies have found that ginsenoside Rb1 can significantly alleviate stress cardiomyopathy (SCM) induced by an acute surge in catecholamines by inhibiting STING-mediated macrophage activation. This finding provides a theoretical basis for its application in stress-induced myocardial injury [192]. Furthermore, β-sitosterol (SITO), a plant sterol widely distributed in plants, exhibits multiple pharmacological effects, including the suppression of vascular smooth muscle cell proliferation, anti-inflammatory, and antioxidant actions [193]. Recent studies have shown that SITO not only inhibits the proliferation of pulmonary artery smooth muscle cells (PASMCs) by suppressing the expression of proliferating cell nuclear antigen (PCNA), but also regulates the phenotypic transformation of PASMCs through modulation of the cGAS/STING signaling pathway. This contributes to the effective inhibition of pulmonary vascular remodeling associated with pulmonary hypertension (PH) [194]. Based on the current body of research, traditional Chinese medicines and their active monomer components show immense potential in the prevention and treatment of myocardial injury in diabetes-related cardiovascular complications mediated by the cGAS-STING signaling pathway. These natural products may serve as novel alternative therapeutic options as cGAS/STING inhibitors and as adjuncts to non-cGAS/STING inhibition therapies, offering new strategies and choices for clinical treatment of diabetic cardiovascular complications, as summarized in Table 3.

## 6. Conclusions

This review comprehensively elucidates the pivotal role of the cGAS–STING signaling pathway as a molecular hub of immunometabolic regulation, integrating microenvironmental disturbances in diabetes—such as gut microbiota dysbiosis, mitochondrial dysfunction, and epigenetic alterations—to drive the pathogenesis of T2DM-associated cardiovascular complications. We propose a mechanistic cascade: metabolic stress leads to cytosolic DNA leakage (e.g., mtDNA or bacterial DNA), excessive activation of the cGAS–STING pathway, and ultimately cardiovascular damage. This process involves spatiotemporal variations in STING signaling across cardiomyocytes, endothelial cells, macrophages, and fibroblasts.

Importantly, cGAS–STING signaling exhibits dual pathophysiological roles. Under homeostatic conditions, basal STING activation supports tissue repair and antimicrobial defense via controlled type I interferon responses [195]. In contrast, under diabetic conditions, persistent metabolic stress and cell injury cause sustained hyperactivation of this pathway, leading to exacerbated sterile inflammation—thereby accelerating the development of cardiomyopathy, atherosclerosis, and ischemia–reperfusion injury (Figure 3).

Preclinical studies have demonstrated the therapeutic potential of targeting this pathway. Small-molecule inhibitors (e.g., RU.521, C-176, H-151, amlexanox) and natural compounds (e.g., ginsenoside Rg1) have shown efficacy in mitigating inflammation and improving cardiovascular outcomes. However, while cGAS/STING degraders (e.g., TH35, UNC9036) can reduce cGAS/STING expression and attenuate inflammation, their specific effects on the cardiovascular system remain unexplored. Of particular note, the first oral cGAS inhibitor, VENT-03, has entered clinical development, pioneering a new therapeutic direction for cardiometabolic diseases. Nonetheless, clinical translation faces significant challenges: (1) chronic inhibition may impair host antimicrobial defense; (2) the ubiquitous expression of this pathway hinders tissue-specific drug delivery; (3) the optimal therapeutic window in diabetic populations remains undefined.

Several knowledge gaps and controversies remain unresolved. For instance, the role of the noncanonical STING–PERK axis in vascular remodeling requires further clarification; the bidirectional modulation of STING by gut microbes (e.g., *Akkermansia muciniphila*) varies across cell types; and not all studies agree on the benefits of cGAS–STING inhibition in diabetic cardiomyopathy or atherosclerosis. Future research should focus on (1) developing organ-selective modulators to reduce off-target immunosuppression, (2) exploring combination therapies such as cGAS/STING inhibitors with GLP-1 receptor agonists, and (3) validating circulating mtDNA or 2′,3′-cGAMP as biomarkers for patient stratification and therapeutic monitoring.

In conclusion, the cGAS–STING axis serves both as a pathological driver and a potential protective modulator in diabetes-related cardiovascular diseases. Unraveling its spatiotemporal regulation, cell-specific effects, and inter-individual variability will be crucial for advancing precision-targeted therapies and successful clinical translation.

## Figures and Tables

**Table 1:** Therapeutic Agents Targeting the cGAS-STING Signaling Pathway.

Target	Medicines Vocabularies	Molecular Mechanism	Ref
cGAS	Aspirin	Enhancing the acetylation of cGAS at lysine residues 384, 394, and 414 effectively inhibits the activity of cGAS.	[140]
cGAS	A151, Suramin, HCQ, QC, X6	Competitively binds to cGAS, preventing the interaction between dsDNA and cGAS.	[141,142]
cGAS	RU. 521, G150, G108, PF-06928215, VENT-03 (compound 25)	Binding to key residues in the catalytic site of cGAS to reduce the binding of cGAS to ATP/GTP.	[145,146,148]
STING	Astin C, SN-011 Gelsevirine	Targeting the CDN binding pocket of STING to block CDN binding.	[152,153,154]
STING	C-176, C-178, C-170, C-171, H-151, CXA-10	Targeting Cys91 on STING to block activation-induced palmitoylation.	[136,155,157]
STING	Tetradroisoquinolone acetic acid (compound 18)	Binding to the cGAMP binding site, thereby displacing the cGAMP binding site on STING.	[158]
STING	The cyclin-dependent protein kinase (CDK) inhibitor 29 (palbociclib)	Directly binds to STING and targets the Y167 residue to block its dimerization and translocation.	[159]
TBK1	Amlexanox	Inhibiting the phosphorylation of STING at Ser366 induced by TBK1 to block the full activation of STING.	[160]
TBK1	BX795, MRT67307, GSK8612, SU6668	Competitively occupying its ATP-binding pocket, thereby preventing ATP access and abrogating kinase activity	[165,166,167,168]

**Table 2:** Degraders Related to the cGAS-STING Signaling Pathway.

Target	Medicines Vocabularies	Molecular Mechanism	Ref
cGAS	Cullin-RING Ligase 5	Targets nuclear cGAS for ubiquitination and degradation through the CRL5 E3 ligase complex.	[171]
cGAS	TH35(PROTAC)	Recruits CRBN E3 ligase via PROTAC to induce ubiquitination and proteasomal degradation of cGAS.	[172]
2′3′-cGAMP	ENPP1	Degrades 2′3′-cGAMP through phosphodiesterase activity, thereby blocking downstream STING activation.	[174]
2′3′-cGAMP	SMPDL3A	Hydrolyzes 2′3′-cGAMP under regulation of lipid metabolic signals to inhibit innate immune activation.	[175]
STING	RNF5/TRIM30a/TRIM29	Catalyzes K48-linked ubiquitination of STING, targeting it for degradation via the proteasome pathway.	[176,177,178]
STING	ESCRT	Promotes K63-linked ubiquitination at Lys288, triggering ESCRT-mediated microautophagy of STING.	[179]
STING	HRD1	Regulates homeostasis of nascent STING through ubiquitin-mediated degradation in the endoplasmic reticulum.	[180]
STING	UNC9036	Activates and phosphorylates STING, then recruits VHL E3 ligase to induce its proteasomal degradation.	[181]
TBK1	3i (PROTAC)	Links a TBK1-targeting ligand with a VHL ligand to induce ubiquitination and potent proteasomal degradation.	[182]
TBK1	degrader 30 (molecular glue)	Recruits RNF126 E3 ligase via molecular glue strategy to promote TBK1 degradation.	[183]

**Table 3:** Modulation of the cGAS-STING Pathway by Herbal Compounds and Monomers: Therapeutic Potential and Mechanisms.

Drug Name	Animal Type	Animal Disease Model	Drug Effect	Biomarker Changes	Ref
MLNO	Wistar Rats	TAO	Inflammation ↓ Coagulation ↓	IL-1β, IL-6, TNF-α, CCL2, PAI-1, TF, ICAM-1, VCAM-1 ↓	[185]
As-IV and Ta-IIA	C57BL/6 J mice	MI/RI	Inflammation ↓ Oxidative stress ↓ Myocardial Function ↑	GSH; SOD ↑ CK, CKMB, LDH, MDA, IL-6, IL-1β, TNF-α ↓	[188]
TET	ApoE−/− mice	AS	Inflammation ↓ atherosclerotic plaque ↓	Ccl2, TNF-α, IL-6 ↓	[190]
Ginsenoside Rb1	C57BL/6J mice	SCM	Inflammation ↓ Myocardial Function ↑	cTnI, IL-6, IL-1β, CCL2 ↓	[192]
SITO	SD rats	PH	Pulmonary Artery Pressure ↓ Myocardial Function ↑	BAX ↑ PCNA, Bcl-2, γ-H2AX ↓	[194]

Note: ↑ indicates upregulation; ↓ indicates downregulation.
